# Impacts of Chronic Habitat Fragmentation on Genetic Diversity of Natural Populations of *Prunus persica* in China

**DOI:** 10.3390/plants11111458

**Published:** 2022-05-30

**Authors:** Quan Jiang, Qiang Xu, Junfeng Pan, Xiaohong Yao, Zhongping Cheng

**Affiliations:** 1 Key Laboratory of Plant Germplasm Enhancement and Specialty Agriculture, Wuhan Botanical Garden, The Chinese Academy of Sciences, Wuhan 430074, China; jiangquan@wbgcas.cn (Q.J.); panjfaau@163.com (J.P.); 2 College of Life Sciences, University of Chinese Academy of Sciences, Beijing 100049, China; 3 Shangrao Vocational and Technical College, Shangrao 334109, China; xq3217@126.com

**Keywords:** *Prunus persica*, wild population, microsatellites, habitat fragmentation, genetic diversity

## Abstract

Wild peach is an important resource for improving existing peach varieties. However, the extant populations of wild peach show fragmented distribution due to human disturbance and geographic isolation. In this study, we used natural populations (or wild populations) of *Prunus persica* (Rosaceae) to assess the genetic effects of habitat fragmentation. A total of 368 individuals sampled from 16 natural populations were analyzed using 23 polymorphic simple sequence repeat (SSR) markers. *Prunus persica* maintained low within-population genetic variation and high level of genetic differentiation. Two genetic clusters were revealed based on three different methods (UPGMA, PCoA, and STRUCTURE). All populations showed a significant heterozygosity deficiency and most extant populations experienced recent reduction in population size. A significant isolation by distance (IBD) was observed with Mantel’s test. Compared to historical gene flow, contemporary gene flow was restricted among the studied populations, suggesting a decrease in gene flow due to habitat fragmentation. Habitat fragmentation has impacted population genetic variation and genetic structure of *P. persica*. For breeding and conservation purpose, collecting as many individuals as possible from multiple populations to maximize genetic diversity was recommended during the process of germplasm collection. In addition, populations from central China had higher genetic diversity, suggesting these populations should be given priority for conservation and germplasm collection.

## 1. Introduction

Habitat fragmentation caused by anthropogenic activities is considered to reduce evolutionary potential and adversely affect the species’ survival by increasing inbreeding [1,2]. Accumulated evidence has revealed that habitat fragmentation usually eroded within-population genetic variation and increased population genetic differentiation due to genetic drift, founder effects, elevated inbreeding, and restricted inter-population gene flow [3]. However, relatively high levels of genetic diversity [4,5,6] and weak population structure were also observed in several studies [7,8]. Species life traits such as species longevity, mating system, and gene flow patterns, etc. affect the species’ genetic responses to habitat fragmentation [1]. Thus, genetic effects of habitat fragmentation are not likely to be universal and complex [6,9]. More case studies are needed to investigate the genetic effects of habitat fragmentation.

Peach (*Prunus persica* (L.) Batsch) is an important stone crop in temperate climates due to the exotic taste and vibrant color of its fruits, and is now cultivated in temperate regions located between latitudes from 30° to 45° throughout in the world. Peach belongs to the family Rosaceae and originated in China over 2 million years ago [10]. In China, peach has been cultivated for over thousands of years [11,12]. More than 5000 cultivars were developed in the word and over 1000 cultivars in China [13]. Both cultivars (including landraces) and rootstocks for cultivation of peach originated from domestication of wild peach germplasm. China is rich in peach germplasm resources, by which wild peach are widely distributed and have lived in different environments with long histories. Wild peach has been used to cope with the narrow genetic diversity of cultivated cultivars and has greatly contributed to peach industry production [13]. However, wild peach populations have faced many challenges in terms of natural growing environments, global climate change, natural disasters, crops replantation, and land development, which have a serious impact on the survival of wild peach trees. Thus, many extant populations of *P. persica* have been fragmented in China [14,15]. Therefore, *P. persica* is a good model species to reveal the genetic effects of habitat fragmentation.

*Prunus persica* (2*n* = 16) is a perennial plant with a life span of over fifty years [14]. It has a small genome size (230 Mb). The juvenile stage of *P. persica* is about 2–4 years and is shorter than that of other perennial fruit species. The flowering periods of *P. persica* are from late February to early March. Edible fruits contain one seed and mature from June to September [14]. The seeds of peach are spread mainly by gravity and occasionally by animals.

Information about genetic diversity and genetic structure of fruit species may help us to understand their demographic history and evolutionary potential, which is of great importance for designing breeding and conservation programs. There are increasing numbers of reports on evaluation of genetic diversity for peach cultivars, rootstocks, and a limited number of wild individuals based on different types of markers [16,17]. However, studies about the patterns of genetic variation in wild populations of *P. persica* at population level are scarce [16]. Microsatellite markers have been employed intensively in genetic diversity analysis due to their codominance, rich polymorphism, high genome coverage, high reliability, and versatile platforms for genotyping [18,19]. The abundant screened microsatellite markers for peach allowed us to investigate genetic variation and population differentiation of *P. persica* sampled from their entire geographic range [20].

In the present study, we investigated genetic variation and population structure of 16 wild populations with 23 non-tightly-linked SSRs that cover all eight linkage groups of the peach genome. The goal of this study was to investigate the genetic consequences of habitat fragmentation. Specifically, we first quantified the genetic diversity at both population and species levels and compared the result with its related species by means of nuclear microsatellite genotypic data. We then compared the historical gene flow and current gene flow to infer whether habitat fragmentation has impacted gene exchange among populations. The results of this study provide insights into the evolutionary process as well as devising optimum strategies for management of genetic resources and conservation of *P. persica*. Moreover, our study forms an integrated genetic diversity evaluation system of peach including cultivars, landraces, rootstocks, and wild populations by combining with our previous studies [21,22].

## 2. Results

### 2.1. Characteristics of 23 nSSR Loci

The number of alleles per locus (*A*) ranged from 3 to 12, with a total of 149 alleles detected overall. The observed heterozygosity (*H*_O_) ranged from 0 to 0.804, with an average value of 0.185. The expected heterozygosity (*H*_S_) among loci ranged from 0.150 to 0.629, with a mean value of 0.444. The total genetic diversity over all populations (*H*_T_) for each locus ranged from 0.260 to 0.864, with averaged value of 0.638 (Table S1). Significant linkage disequilibrium between any pairs of SSR loci across populations was detected for 229 out of 4048 comparisons (*p* < 0.05), but none of those SSR linkage disequilibria were significant where Bonferroni adjustment was applied.

### 2.2. Genetic Diversity

Genotypic linkage disequilibrium between all loci showed no significant deviation from zero after a Bonferroni adjustment was applied. Across all populations, the allelic richness per population (*A*) ranged from 2.0 to 4.3. The observed heterozygosity (*H*_O_) per population ranged from 0.083 to 0.325, with a mean value of 0.185. The expected heterozygosity (*H*_E_) per population was 0.140 to 0.569, with a mean value of 0.442. All population showed significant deviation from Hardy–Weinberg equilibrium (*p* < 0.05) when all loci were combined. For each population, the inbreeding coefficient (*F*_IS_) ranged from 0.402 to 0.765 (Table 1).

### 2.3. Population Structure

**Figure 1:**
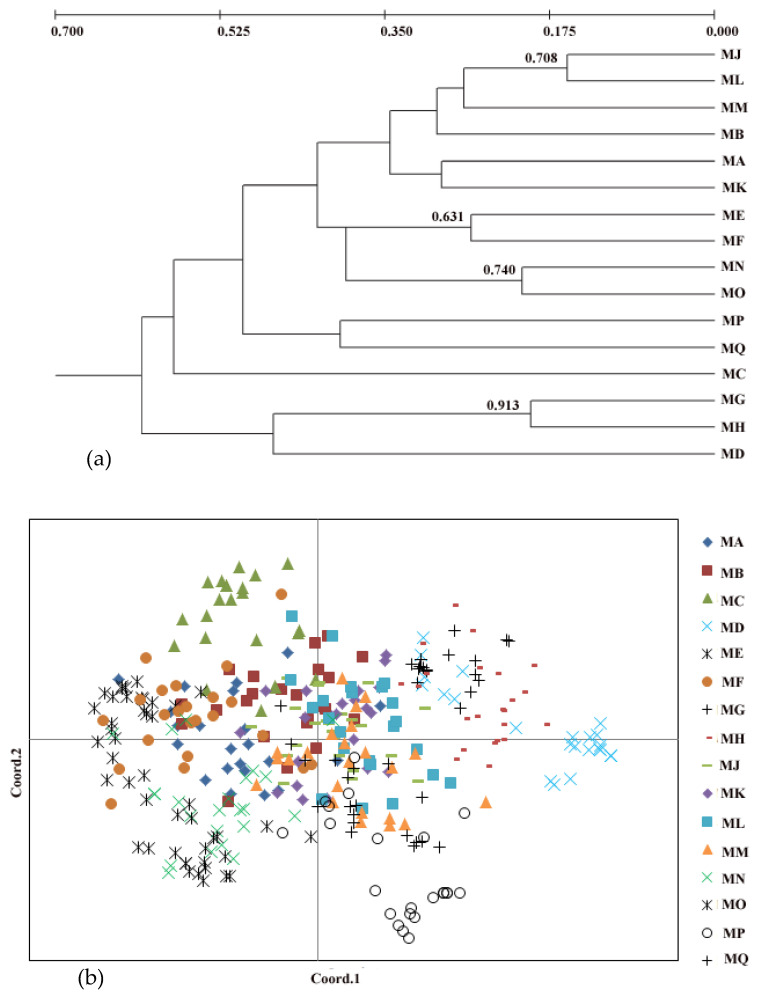
(**a**) Genetic relationship of 16 populations of *P. persica* with the UPGMA dendrogram: bootstrap percentage (>50%) are given above branches; (**b**) principal coordinates analysis for 16 populations.

**Figure 2:**
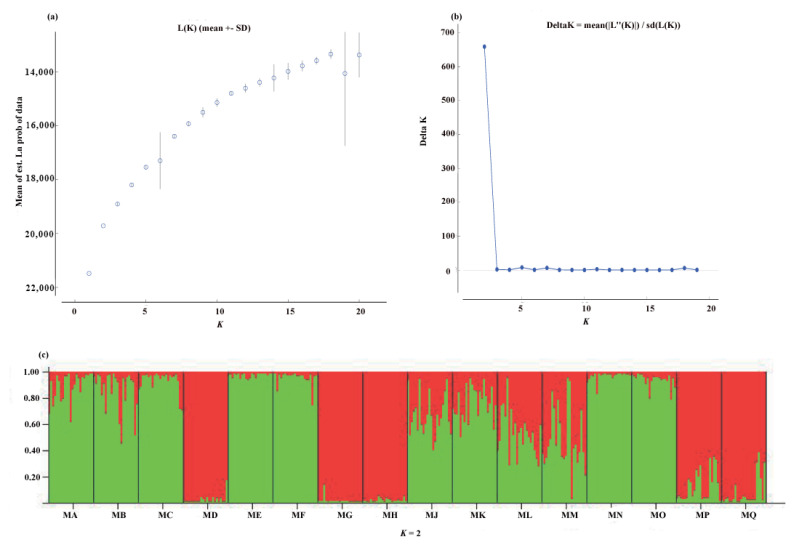
STRUCTURE analysis for the 16 population of *P. persica*: (**a**) mean of log-likelihood values [*L*(*K*)] for each value of *K* in *P. persica*; (**b**) the true *K* values determined using the Δ*K* method; (**c**) assignment of all individuals into two genetic clusters based on the STRUCTURE.

Our UPGMA analysis showed the existence of two genetic clusters. The first-diverging cluster contained three populations (MD, MG, and MH). The second cluster was composed of the remaining 13 populations (Figure 1a). The pattern of genetic clusters was further confirmed by the PCoA analysis (Figure 1b). It was hard to obtain the true *K* value based on the methods of [ln *P*(D)] (mean estimated logarithm of probability) as the [ln *P*(D)] never reached a plateau. The highest peak in Δ*K* at *K* = 2 indicated that two genetic clusters were detected in *P. persica* (Figure 2).

The results of AMOVA testing were presented in Table 2. Of the total genetic diversity, about 32.1% was attributable to divergence among populations, 39.45% to divergence among individuals, and 28.45% resided within individuals. The *F*_ST_ value for wild populations of *P. persica* was 0.321. Pairwise comparisons of genetic differentiation ranged from 0.123 to 0.601, with a mean value of 0.320 (Table S2).

**Figure 3:**
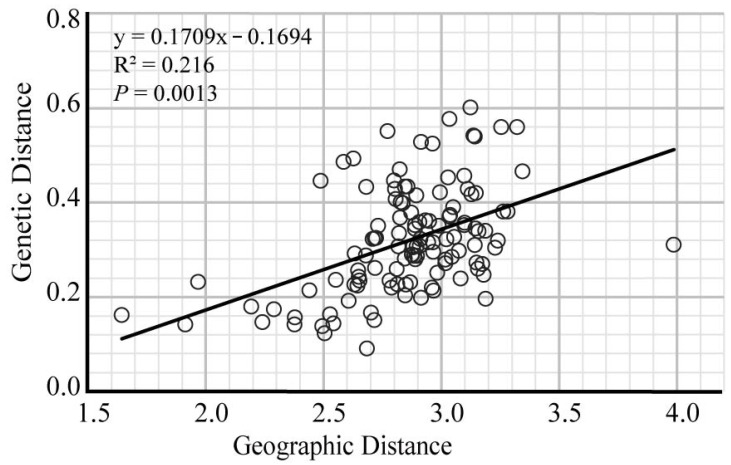
Scatterplots of genetic distances vs. geographical distance among populations of *P. persica*.

The result of Mantel’s test revealed a significant correlation between genetic distances and geographical distances (Mantel’s test, *r*^2^ = 0.216, *p* < 0.05, Figure 3).

### 2.4. Mutation–Drift Equilibrium

According to IAM, a demographic bottleneck in 15 populations except population MO (Table 3) was observed with the Wilcoxon’s statistical test. However, only one (MG) and five populations (MA, MG, MJ, MM, and MQ) showed evidence of a decline of population size under the TPM and SMM model, respectively. All populations showed L-shaped allelic distributions of allele frequencies (Table 3).

### 2.5. Historical Gene Flow vs. Contemporary Gene Flow

Historical gene flow among populations (*m*_h_) ranged from 0.0053 to 0.2327, with a mean value of 0.0923. Higher gene flow from population MK to population MO (0.2197) and from population MN to population MF (0.2327) was observed unidirectionally. BayesAss yielded a relatively low level of contemporary gene flow (*m*_c_), ranged from 0.0085 to 0.0422, with a mean value of 0.0105 (Table S3).

## 3. Discussion

### 3.1. Genetic Diversity of Wild Populations

Although there is much prior research on the genetic variation of peach landraces and cultivars [22,23,24], to the best of our knowledge, our study represents the first effort to address genetic diversity of extant wild populations of *P. persica* using microsatellite markers. It is preferable to benchmark the genetic variation found in this species by conducting comparative studies with a closely related species using the same type of molecular markers. In terms of allele number, the average number of alleles per locus (*A*) was 3.2, which was lower than the 4.5 reported by Testolin et al. [25], the 4.62 observed by Khadivi-Khub et al. [26], and the 6.09 revealed in Cao et al. [24], but higher than the 3.0 revealed by Sosinski et al. [27]. The genetic diversity parameters (*H*_E_ = 0.140–0.569, mean = 0.442) revealed in the present study were lower than those of *Prunus fruticosa* Pall. (*H*_E_ = 0.531–0.735, mean = 0.641 [28]), *Prunus davidiana* (Carrie′re) Franch (*H*_E_ = 0.061–0.868, mean = 0.583 [21]). The above analysis indicates the extant wild populations of *P. persica* maintain a relatively low level of genetic diversity. The low level of genetic diversity for *P. persica* was also revealed in other previous studies using different types of molecular markers [17,29].

The relatively low level of genetic variation revealed in *P. persica* compared to related species such as apricot, almond, and plum can be understood by their different mating system. Peach is self-pollinating and inbreeding, whereas plum, apricot, and almond are generally self-incompatible and outcrossing [30]. In addition, the diversity parameter (*H*_E_ = 0.140–0.569, mean = 0.442) was lower than that of previous work on peach landrace and cultivars with microsatellite markers (*H*_E_ = 0.03–0.85, mean = 0.607 [31]), which suggests significant effects of habitat fragmentation on the genetic variation of *P. persica*, in line with theoretical expectations. Reduced variability after fragmentation has been also found in small populations of other plants such as *Cariniana estrellensis* [32]. Our field investigation showed population sizes of most populations of wild peach were less than 100 individuals [15]. The low population size due to habitat fragmentation would result in low levels of genetic diversity at the population level [2].

The narrow genetic base of peach has resulted in low variability within the species [33]. The low level of genetic diversity may indicate potential issues with breeding depression. In the present study, the *H*_O_ values of all SSR loci were much lower than the *H*_E_ values, showing a significant heterozygosity deficiency among Chinese wild peach. In addition, with the use of IAM model, most of the extant populations of *P. persica* have likely experienced a recent population bottleneck. Thus, persistent habitat fragmentation will jeopardize the long-term survival of *P. persica* through increasing inbreeding and genetic drift.

### 3.2. Genetic Structure

Accumulated evidence has demonstrated that habitat fragmentation has great impacts on the population differentiation, i.e., fragmented populations have increased genetic structure [1]. In the present study, some evidence indicated strong genetic structure in *P. persica*. First, the overall *F*_ST_ value (0.320) indicated high levels of genetic differentiation between populations (Table S2). Second, the AMOVA analysis revealed that 32.1% of the total genetic diversity was attributed to among populations (Table 2). In addition, significant correlation between genetic and geographic distances suggests restricted gene flow among the extant populations due to geographic isolation, which in turn would give rise to high genetic differentiation (Figure 3). Therefore, we speculate that habitat fragmentation caused by human activities and geographic factors have impacted the genetic differentiation of wild populations of *P. persica*. A similarly high level of genetic differentiation revealed by microsatellite markers was observed in other plants with similarly fragmented distribution, such as *Ceiba aesculifolia* [34].

Habitat fragmentation destroys the connectivity of populations, hindering gene exchange among populations [1]. The strong population structure among populations is therefore commonly explained as a result of limited gene flow, either mediated by pollen or by seed. In the present study, compared to historical gene flow, contemporary gene flow analysis revealed a relatively low level of genetic exchange among studied populations (Table S3), suggesting that a decrease in gene flow and geographic isolation due to habitat fragmentation may have impeded gene exchange among extant populations of *P. persica*. In our field observation, most of the fruits of the wild populations of *P. persica* fall within the canopy, suggesting that their seeds are dispersed mainly by gravity, although we can not exclude occasional dispersion by various types of animals [15]. Thus, gene flow via seed dispersal may be restricted.

Although peach is considered to have been originally domesticated in China about 4000–5000 years ago [11], the direct ancestral relatives of cultivated peach remain unknown [12]. The UPGMA dendrogram separates the wild populations of *P. persica* into two clusters. Two populations MG and MH from Shandong province and one population MD from Hunan province formed a cluster. The remaining 13 populations were clustered into a group but did not cluster together based on geographical origin. Because the seeds of peach are wrapped in a hard wooden structure, they are easy to spread over long distances. The seed dispersion mediated by human or animal is therefore another explanation for this population structure pattern. The current population structure revealed in the present study suggests the evolutionary pattern of wild peach is very complex, which is also supported by the results of PCoA and STRUCTURE analysis where individuals from different populations were clustered together. Further studies are therefore needed to investigate the demographic history of wild peach using many more populations and molecular markers.

### 3.3. Implication for Genetic Conservation

Crop wild relatives have been used for decades in crop improvement for enhancing plant performance and improving adaptation for current and future climates [35]. As a fruit tree requiring adequate winter chill for growth and development, peach is sensitive to climate change. However, global climate change would decrease winter chill in areas where peach is traditionally cultivated, thereby threatening peach production. Therefore, it is very important to conserve peach wild relatives with wide phenotypic variations in flowering phenology. In addition, wild peach has rich phenotypic variation in fruit size, shape, color, texture, flavor, fruit mass, Vitamin C, soluble solids content, soluble sugars, titratable acidity, fruit development period, date of ripening, etc. The rich diversity in phenotypic variation of wild peach has greatly contributed to the development of modern peach cultivars. Information about genetic variation and population structure is vital to conservation and germplasm collection. In the present study, the results of our investigation indicated that the extant populations of wild peach maintained low levels of genetic variation at the population level and high genetic differentiation. Both historical gene flow and contemporary gene flow were restricted. In addition, all populations showed heterozygote deficiency. The above genetic information suggested all populations should be conserved. For populations with a small population size, ex situ conservation measures and germplasm collection should be urgently implemented [2]. In addition, the allele number and *H*_E_ of Central China (populations MA, MJ, MK, and MM) were higher than those of other populations (Table 1). Therefore, these populations from Central China should be given priority for conservation.

## 4. Materials and Methods

### 4.1. Plant Material

**Figure 4:**
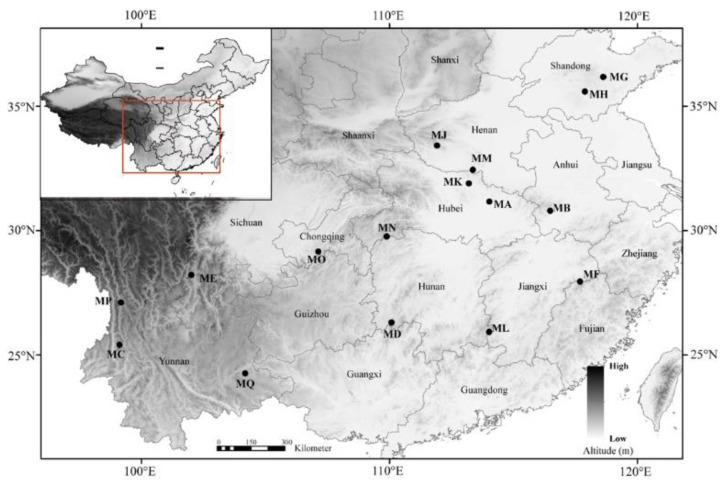
Locations of the 16 populations of *P. persica* sampled for this study. Cool to warm colors represent low to high altitude.

A total of 368 individuals representing 16 populations (23 individuals per population) were sampled across the entire geographic range of *P. persica* (Figure 4 and Table 1). The distance between any two samples was, at minimum, 50 m. Fresh leaves were dried quickly by using silica gel after collection and stored at −20 °C until DNA extractions and genotyping.

### 4.2. Microsatellite Genotyping

Genomic DNA was isolated from approximately 20 mg of dried leaves using the cetyltrimethylammonium bromide (CTAB) method [36]. Quality and DNA concentration were confirmed using Microcolume Spectrophotometer ND5000 (BioTeke, Beijing, China). Twenty-three nuclear SSR markers were used to genotype all samples (Table S4). PCR amplifications of SSR loci and genotyping followed the protocol of Cheng et al. [21]. Fluorescent-labelled PCR products were analyzed on a 3730xl DNA Analyzer (Applied BioSystems, Waltham, MA, USA). MSAnalyser was used to check SSR quality.

### 4.3. Data Analysis

The genetic diversity parameters for each locus, including the observed number of alleles (*A*), observed heterozygosity (*H*_O_), expected heterozygosity (*H*_S_) under Hardy–Weinberg equilibrium (HWE), and total genetic diversity over populations (*H*_T_), were estimated by FSTAT (version 2.9.3) [37]. Linkage disequilibrium between microsatellites were tested by Fisher’ exact tests in GENEPOP 3.4 [38], with 10000 dememorizations, 1000 batches and 10000 iterations. For each population, GenALEx 6.1 [39] was used to estimate the genetic diversity parameters. The estimates include the number of alleles (*A*), observed heterozygosity (*H*_O_), expected heterozygosity (*H*_E_), and fixation indices (*F*_IS_). Departure from Hardy–Weinberg expectations were tested using the default parameters of GENEPOP 3.4.

Wright’s *F*-statistics *F*_ST_ [40] for all populations and all population pairs were estimated with GENEPOP 3.4 in accordance with Weir and Cockerham [41]. Molecular variance (AMOVA) analysis was conducted to determine the partitioning of genetic variation within and among populations using ARLEQUIN 2.0 [42]. An unweighted pair group mean analysis (UPGMA) was conducted by TFPGA [43] with 1000 permuted trees bootstrapped across loci. The genetic distance matrix was estimated by using Nei’s unbiased distance [44].

The hierarchical population structure analysis was implemented using the Bayesian-based program STRUCTURE 2.3.4 [45]. The optimal *K* value was determined by calculating the posterior probability for each mean value of *K* by using the mean log value of the likelihood (Ln*Pr* (X|K)]). An additional determination of the optimal number of populations (*K*) was predicted using Δ*K* parameter [46]. The numbers of populations were set as 2–20. Each run was started with run length of 50000 iterations and a burn-in period of 10^6^, and was replicated 10 times. For the STRUCTURE analysis, the admixture model and uncorrelated allele frequencies were adopted.

The genetic bottleneck test was performed using the program Bottleneck [47]. The significance of heterozygote excess was tested using the Wilcoxon’s sign–rank test under the infinite allele model (IAM), the stepwise mutation model (SMM), and the two-phase model (TPM), according to the methods of Piry et al. [47]. For each population, 10^4^ simulations were performed for each mutational model.

Mantel’s test was used to examine the association between genetic (*F*_ST_) and geographical distances with 999 random permutations in the package Vegan v2.4. Geographical distances were shown by the log10 of straight-line distance between pairs of populations.

The amount of historical gene flow among populations was estimated by calculating the mutation-scaled migration rate (M) with the approximation of standard Brownian motion using Migrate-n v. 3.6 [48] with 500,000 genealogies, a 10,000-genealogy burn-in, and three runs. Immigration rate (m) was calculated as m = *M*μ, where μ is the mutation rate (nSSRs, 3 × 10^−4^ [18]). The contemporary gene flow was calculated using BayesAss v. 3.0 [49] with 5,000,000 MCMC iterations and 20% burn-in.

## 5. Conclusions

In this study, the genetic diversity and genetic structure of 16 wild populations in *P. persica* were assessed by 23 microsatellite markers. Our study revealed that *P. persica* maintained low within-population genetic variation and strong population structure. Two major genetic clusters were detected based three different methods. A significant heterozygosity deficiency was found in all populations and most populations experienced recent reduction in population size. Mantel’s test revealed a significant isolation by distance (IBD) pattern. A decrease in gene flow caused by habitat fragmentation was observed by comparing historical gene flow with contemporary gene flow. Overall, our study showed habitat fragmentation has impacted population genetic variation and genetic structure of *P. persica*. Our results provide insights into peach conservation and germplasm collection.

## Figures and Tables

**Table 1:** Information about of collection sites, sample sizes (*n*), genetic diversity parameters of the 16 natural populations of *Prunus persica*.

Population Code	Population Locality	Altitude (m)	Latitude (N)	Longitude (E)	*N*	*A*	*H*_O_	*H*_E_	*F*_IS_
MA	Xiaogan, Hubei Province	250–500	31°10′	114°03′	23	4.0	0.240	0.590	0.593 **
MK	Suizhou, Hubei Province	800–1025	31°54′	113°13′	23	3.5	0.219	0.502	0.564 **
MJ	Nanyang, Henan Province	450–600	33°25′	111°56′	23	3.8	**0.325**	0.544	**0.402** *
MM	Xingyang, Henan Province	400–600	32°27′	113°23′	23	4.0	0.285	**0.569**	0.498 **
MD	Shaoyang, Hunan Province	450–650	26°18′	110°06′	23	2.5	0.115	0.372	0.690 **
MN	Sangzhi, Hunan Province	530–620	29°46′	109°54′	23	2.6	0.144	0.386	0.628 **
ME	Mianning, Sichuan Province	1910	28°13′	102°01′	23	3.0	0.142	0.409	0.653 **
MG	Qingzhou, Shandong Province	1032	36°11′	118°38′	23	**2.0**	**0.083**	**0.140**	0.407 *
MH	Mengyin, Shandong Province	450	35°36′	117°54′	23	3.2	0.217	0.438	0.504 **
MB	Anqing, Anhui Province	400–650	30°48′	116°30′	23	**4.3**	0.229	0.568	0.597 **
ML	Shangyou, Jiangxi Province	600–650	25°55′	114°02′	23	3.7	0.240	0.523	0.541 **
MF	Qianshan, Jiangxi Province	587	27°57′	117°42′	23	3.7	0.166	0.510	0.674 **
MO	Nanchuan, Chongqing City	730–850	29°09′	107°09′	23	2.8	0.146	0.375	0.612 **
MC	Baoshan, Yunnan Province	1200	25°24′	99°08′	23	2.8	0.091	0.387	**0.765** **
MP	Weixi, Yunnan Province	2100	27°06′	99°11′	23	2.6	0.113	0.381	0.703 **
MQ	Qiubei, Yunnan Province	1580	24°15′	104°12′	23	2.3	0.206	0.376	0.452 **
Average					23	3.2	0.185	0.442	

A, average number of alleles per locus; *H*_E_, expected heterozygosity; *H*_O_, observed heterozygosity; *F*_IS_, within-population coefficient of inbreeding. * *p* < 0.05, ** *p* < 0.01.

**Table 2:** The analysis of molecular variance (AMOVA) for *P. persica* populations.

Source of Variation	d.f.	Sum of Squares	Variance Components	Percentage of Variation	*p*-Value
Among populations	15	1777.571	2.40156 Va	32.1	*p* < 0.001
Among individuals	352	2827.565	2.95189 Vb	39.45	*p* < 0.001
Within individuals	368	783.500	2.12908 Vc	28.45	*p* < 0.001
Total	735	5838.636	7.48253		

The *F*_ST_ value for *P. persica* was 0.321.

**Table 3:** Probabilities for mutation–drift equilibrium in 16 populations of *P. persica* under the three models with the Wilcoxon’s statistical tests. *, *p* < 0.05; **, *p* < 0.01.

Population	Mutation–Drift Test			
	IAM	TPM	SMM	Mode Shift
MA	0.001 **	0.038 *	0.753	L-shaped
MB	0.002 **	0.329	0.052	L-shaped
MC	0.033 *	0.368	0.674	L-shaped
MD	0.002 **	0.064	0.701	L-shaped
ME	0.048 *	0.388	0.841	L-shaped
MF	0.018 *	0.410	0.463	L-shaped
MG	0.039 *	0.016 *	0.006 *	L-shaped
MH	0.026 *	0.257	0.609	L-shaped
MJ	0.001 **	0.016 *	0.975	L-shaped
MK	0.006 **	0.151	0.890	L-shaped
ML	0.004 **	0.079	1.000	L-shaped
MM	0.000 **	0.005 **	0.974	L-shaped
MN	0.024 *	0.216	0.812	L-shaped
MO	0.076	0.701	0.349	L-shaped
MP	0.005 **	0.143	0.956	L-shaped
MQ	0.000 **	0.002 **	0.087	L-shaped

IAM, infinite allele model; TPM, two-phase model; SMM, stepwise mutation model (SMM).

## Data Availability

Not applicable.

## Supplementary Materials

The following supporting information can be downloaded at: https://www.mdpi.com/article/10.3390/plants11111458/s1, Table S1: Characteristics of 23 nSSR loci surveyed across 16 natural populations of *P. persica*; Table S2: Matrix of pairwise comparisons of genetic differentiation (*F*_ST_) for natural populations of *P. persica*; Table S3: Migration rates (m) across the 16 natural populations of *P. persica*. Table S4: Twenty-three SSR markers used for amplification of accessions of 16 natural populations of *P. persica*.
