# Supplementary material for: Impacts of Chronic Habitat Fragmentation on Genetic Diversity of Natural Populations of Prunus persica in China
Source: Plants (Basel). 2022 May 30;11(11):1458. doi: 10.3390/plants11111458 (PMC9183131; doi:10.3390/plants11111458)
Supplement: Supplementary file 1 [file plants-11-01458-s001.zip › plants-1726841-supplementary.pdf]

Table S1. Characteristics of 23 nSSR loci surveyed across 16 natural populations of *P. persica*.

Locus	A	H_O	H_S	H_T
BPPCT 006	6	0.804	0.528	0.579
BPPCT 007	5	0.014	0.338	0.584
BPPCT 008	9	0.144	0.568	0.798
BPPCT 015	8	0.082	0.387	0.638
BPPCT 017	6	0.215	0.465	0.709
BPPCT 020	9	0.625	0.477	0.590
BPPCT 025	6	0.087	0.418	0.572
BPPCT 028	6	0.035	0.405	0.637
CPPCT 002	4	0.766	0.552	0.654
CPPCT 006	4	0.035	0.447	0.683
CPPCT 016	3	0.038	0.150	0.260
CPPCT 017	5	0.155	0.522	0.680
CPPCT 022	5	0.090	0.499	0.724
CPPCT 033	4	0.000	0.353	0.511
UDP96-001	8	0.250	0.576	0.704
UDP96-003	10	0.245	0.629	0.814
UDP96-005	6	0.163	0.370	0.596
UDP96-013	6	0.114	0.363	0.695
UDP97-403	6	0.027	0.437	0.733
UDP98-024	9	0.160	0.461	0.622
UDP98-025	4	0.014	0.156	0.305
UDP98-409	12	0.122	0.613	0.864
UDP98-412	8	0.073	0.497	0.712
Average	6.5	0.185	0.444	0.638

Note: A, number of alleles per locus; H_O, observed heterozygosity within populations; H_S, expected heterozygosity within populations; H_T, total genetic diversity over all populations.

Table S2. Matrix of pairwise comparisons of genetic differentiation (F_{ST}) for natural population of *P. persica*.

	MA	MB	MC	MD	ME	MF	MG	MH	MJ	MK	ML	MM	MN	MO	MP	MQ
MA	-	**	**	**	**	**	**	**	**	**	**	**	**	**	**	**
MB	0.143	-	**	**	**	**	**	**	**	**	**	**	**	**	**	**
MC	0.226	0.288	-	**	**	**	**	**	**	**	**	**	**	**	**	**
MD	0.368	0.359	0.457	-	**	**	**	**	**	**	**	**	**	**	**	**
ME	0.239	0.260	0.381	0.528	-	**	**	**	**	**	**	**	**	**	**	**
MF	0.167	0.163	0.325	0.415	0.196	-	**	**	**	**	**	**	**	**	**	**
MG	0.434	0.447	0.551	0.542	0.560	0.525	-	**	**	**	**	**	**	**	**	**
MH	0.289	0.251	0.417	0.322	0.422	0.357	0.311	-	**	**	**	**	**	**	**	**
MJ	**0.123**	0.151	0.296	0.308	0.285	0.198	0.399	0.256	-	**	*	**	**	**	**	**
MK	0.157	0.224	0.362	0.352	0.327	0.231	0.470	0.261	0.142	-	**	**	**	**	**	**
ML	0.174	0.162	0.324	0.280	0.310	0.236	0.407	0.213	0.091	0.192	-	**	**	**	**	**
MM	0.180	0.144	0.344	0.305	0.298	0.227	0.429	0.220	0.147	0.232	0.138	-	**	**	**	**
MN	0.226	0.259	0.390	0.486	0.307	0.287	0.577	0.401	0.235	0.292	0.235	0.243	-	**	**	**
MO	0.203	0.316	0.420	0.493	0.323	0.279	**0.601**	0.434	0.307	0.335	0.316	0.281	0.214	-	**	**
MP	0.247	0.319	0.466	0.370	0.446	0.381	0.560	0.352	0.273	0.339	0.304	0.270	0.374	0.323	-	**
MQ	0.287	0.307	0.453	0.433	0.429	0.351	0.540	0.345	0.271	0.351	0.290	0.220	0.379	0.361	0.340	-

**, $P < 0.01$, *, $P < 0.05$.

Table S3. Migration rates (m) across the 16 natural populations of *P. persica*.

	MA	MB	MC	MD	ME	MF	MG	MH	MJ	MK	ML	MM	MN	MO	MP	MQ
Mean historical migration rates																
MA		0.0915	0.1111	0.0543	0.0775	0.1311	0.0051	0.0177	0.0409	0.0655	0.0899	0.1825	0.0765	0.2089	0.0719	0.0787
MB	0.0735		0.0213	0.1475	0.0479	0.0467	0.0387	0.1435	0.1001	0.0861	0.2171	0.1271	0.0931	0.1629	0.0193	0.0159
MC	0.1527	0.0903		0.0703	0.1631	0.1143	0.0717	0.0409	0.1643	0.0875	0.0603	0.1491	0.0473	0.0455	0.0497	0.0047
MD	0.0719	0.0907	0.1059	0.0000	0.1411	0.0803	0.0879	0.0635	0.1267	0.0261	0.1049	0.0817	0.1221	0.1887	0.1455	0.1797
ME	0.0123	0.0121	0.0291	0.0327	0.0000	0.1483	0.0129	0.1431	0.0765	0.1541	0.1045	0.1291	0.1261	0.1531	0.0693	0.0409
MF	0.0161	0.0089	0.0317	0.1397	0.0613		0.1181	0.0601	0.1887	0.0053	0.1101	0.1197	0.0415	0.1103	0.0659	0.0141
MG	0.1467	0.0695	0.0459	0.1160	0.0581	0.0133		0.0415	0.1175	0.1217	0.2229	0.0777	0.1049	0.0593	0.0681	0.1137
MH	0.0105	0.0665	0.0231	0.1325	0.0719	0.1007	0.1585		0.1503	0.1473	0.0119	0.1153	0.0457	0.1121	0.0789	0.2911
MJ	0.0915	0.1049	0.0169	0.0291	0.1415	0.1779	0.2493	0.1041		0.1759	0.1021	0.0545	0.0919	0.0881	0.0105	0.0063
MK	0.0855	0.1417	0.0469	0.0467	0.0685	0.0815	0.0519	0.0363	0.0679		0.1519	0.0443	0.1543	0.1129	0.0793	0.0269
ML	0.0085	0.0703	0.0369	0.1461	0.0075	0.0617	0.0755	0.1913	0.2443	0.0079		0.1817	0.0677	0.0839	0.1635	0.0065
MM	0.0443	0.1257	0.1059	0.0555	0.1635	0.1069	0.0645	0.0633	0.2165	0.0827	0.1839		0.0963	0.0583	0.0581	0.1723
MN	0.0885	0.0601	0.0751	0.0767	0.0733	0.2327	0.0641	0.1265	0.0633	0.1373	0.1707	0.0729		0.1401	0.0299	0.0915
MO	0.0575	0.0267	0.1273	0.0055	0.0597	0.0355	0.1053	0.0695	0.0727	0.2197	0.0659	0.1427	0.0697		0.1205	0.0157
MP	0.1541	0.2543	0.0869	0.1365	0.0733	0.0777	0.1021	0.1937	0.0789	0.1231	0.0697	0.1187	0.1075	0.0931		0.1151
MQ	0.0967	0.1797	0.0357	0.0891	0.0543	0.0103	0.0387	0.1217	0.0073	0.2169	0.2301	0.1829	0.0915	0.0659	0.0371	
Mean recent migration rate																
MA		0.0098	0.0115	0.0087	0.0090	0.0089	0.0087	0.0086	0.0094	0.0168	0.0089	0.0422	0.0088	0.0177	0.0086	0.0088
MB	0.0210		0.0093	0.0085	0.0098	0.0100	0.0086	0.0086	0.0174	0.0122	0.0128	0.0090	0.0096	0.0087	0.0085	0.0087
MC	0.0095	0.0107		0.0126	0.0087	0.0098	0.0085	0.0086	0.0095	0.0116	0.0105	0.0096	0.0092	0.0091	0.0085	0.0086
MD	0.0086	0.0085	0.0096		0.0086	0.0111	0.0085	0.0086	0.0102	0.0138	0.0085	0.0135	0.0086	0.0086	0.0087	0.0085
ME	0.0110	0.0093	0.0086	0.0085		0.0087	0.0086	0.0086	0.0087	0.0086	0.0086	0.0089	0.0085	0.0089	0.0086	0.0086
MF	0.0147	0.0131	0.0100	0.0086	0.0168		0.0086	0.0087	0.0088	0.0099	0.0090	0.0086	0.0087	0.0087	0.0086	0.0086
MG	0.0093	0.0085	0.0086	0.0085	0.0088	0.0086		0.0106	0.0088	0.0086	0.0120	0.0085	0.0085	0.0086	0.0097	0.0086
MH	0.0092	0.0085	0.0086	0.0086	0.0086	0.0085	0.0157		0.0096	0.0085	0.0088	0.0086	0.0085	0.0086	0.0115	0.0085
MJ	0.0098	0.0106	0.0086	0.0089	0.0088	0.0087	0.0088	0.0085		0.0128	0.0405	0.0118	0.0088	0.0109	0.0086	0.0089
MK	0.0104	0.0105	0.0090	0.0094	0.0085	0.0125	0.0085	0.0086	0.0115		0.0165	0.0139	0.0088	0.0123	0.0092	0.0087
ML	0.0100	0.0154	0.0104	0.0086	0.0101	0.0094	0.0111	0.0085	0.0099	0.0089		0.0458	0.0087	0.0093	0.0088	0.0088
MM	0.0144	0.0090	0.0098	0.0087	0.0088	0.0094	0.0086	0.0092	0.0092	0.0089	0.0099		0.0087	0.0092	0.0089	0.0088
MN	0.0166	0.0225	0.0162	0.0088	0.0086	0.0099	0.0085	0.0086	0.0107	0.0125	0.0130	0.0103		0.0108	0.0086	0.0086

MO	0.0258	0.0090	0.0108	0.0087	0.0096	0.0087	0.0086	0.0085	0.0148	0.0128	0.0087	0.0096	0.0092		0.0086	0.0087
MP	0.0089	0.0086	0.0086	0.0086	0.0085	0.0085	0.0088	0.0134	0.0147	0.0091	0.0096	0.0176	0.0085	0.0092		0.0087
MQ	0.0092	0.0089	0.0089	0.0087	0.0085	0.0086	0.0087	0.0085	0.0119	0.0089	0.0139	0.0093	0.0086	0.0087	0.0084	

The source populations for migration are given in rows and populations receiving migrants are in columns. m = $M\mu$ (M is the mutation–scaled effective immigration rate, μ is the mutation rate per site: 3×10^{-4}).

Table S4. Twenty-three SSR markers used for amplification of 16 populations of wild *P. persica*.

Marker	Linkage group	Forward sequence	Reverse sequence	Reference
BPPCT 006	G8	GCTTGTGGCATGGAAGC	CCCTGTTTCTCATAGAACTCACAT	Dirlewanger et al. 2002
BPPCT 007	G3	TCATTGCTCGTCATCAGC	CAGATTTCTGAAGTTAGCGGTA	
BPPCT 008	G6	ATGGTGTGTATGGACATGATGA	CCTCAACCTAAGACACCTTCACT	
BPPCT 015	G4	ATGGAAGGGAAGAGAAATCG	GTCATCTCAGTCAACTTTTCCG	
BPPCT 017	G5	TTAAGAGTTTGTGATGGGAACC	AAGCATAATTTAGCATAACCAAGC	
BPPCT 020	G1	CGTGGATGGTCAAGATGC	ATTGACGTGGACTTACAGGTG	
BPPCT 025	G6	TCCTGCGTAGAAGAAGGTAGC	CGACATAAAGTCCAAATGGC	
BPPCT 028	G1	TCAAGTTAGCTGAGGATCGC	GAGCTTGCCTATGAGAAGACC	
CPPCT 002	G3	GGAGCTGCAATATTGCTG	GTTAGGGAAGCATCTCAC	Aranzana et al. 2003
CPPCT 006	G8	AATTAACTCCAACAGCTCCA	ATGGTTGCTTAATTCAATGG	
CPPCT 016	G1	AATTCCCTATGGAAATTAGA	CGCATATTATAGGTAGGAAA	
CPPCT 017	G7	TGACATGCATGCACTAAACAA	TGCAAATGCAATTTCATAAAGG	
CPPCT 022	G7	CAATTAGCTAGAGAGAATTATTG	GACAAGAAGCAAGTAGTTTG	
CPPCT 033	G7	TCAGCAAACTAGAAACAAACC	TTGCAATCTGGTTGATGTT	
UDP96-001	G6	AGTTTGATTTTCTGATGCATCC	TGCCATAAGGACCGGTATGT	Cipriani et al. 1999
UDP96-003	G4	TTGCTCAAAAGTGTCGTTGC	ACACGTAGTGCAACACTGGC	Testolin et al. 2000
UDP96-005	G1	GTAACGCTCGCTACCACAAA	CCTGCATATCACCACCCAG	
UDP96-013	G2	ATTCTTCACTACACGTGCACG	CCCCAGACATACTGTGGCTT	
UDP97-403	G3	CTGGCTTACAACTCGCAAGC	CGTCGACCAACTGAGACTCA	
UDP98-024	G4	CCTTGATGCATAATCAAACAGC	GGACACACTGGCATGTGAAG	
UDP98-025	G2	GGGAGGTTACTATGCCATGAAG	CGCAGACATGTAGTAGGACCTC	
UDP98-409	G8	GCTGATGGGTTTTATGGTTTTC	CGGACTCTTATCCTCTATCAACA	
UDP98-412	G6	AGGGAAAGTTTCTGCTGCAC	GCTGAAGACGACGATGATGA	
